# A Theoretical Study of Clorsulon-Imprinted Polypyrrole: Modeling Complementary Cavity Formation and Rebinding of Clorsulon

**DOI:** 10.1021/acsmeasuresciau.6c00052

**Published:** 2026-05-08

**Authors:** Enayat Mohsenzadeh, Vilma Ratautaite, Agne Ramanaviciute, Arunas Ramanavicius

**Affiliations:** † Department of Nanotechnology, State Research Institute Centre for Physical Sciences and Technology (FTMC), Sauletekio Ave. 3, LT-10257 Vilnius, Lithuania; ‡ Department of Physical Chemistry, Institute of Chemistry, Faculty of Chemistry and Geosciences, Vilnius University (VU), Naugarduko Str. 24, LT-03225 Vilnius, Lithuania; § Department of Physics, University of Cambridge, JJ Thomson Avenue, Cambridge CB3 0HE, U.K.

**Keywords:** density functional theory (DFT), clorsulon, molecularly imprinted polymer (MIP), polypyrrole (PPy), metadynamics

## Abstract

Clorsulon is highly toxic to aquatic organisms, including fish and invertebrates, and can have long-term adverse effects on the environment. It is commonly used in cattle, and residues excreted in dung can negatively affect dung-dependent insects. Direct contamination of waterways, such as ponds, streams, or ditches, should be strictly avoided. Therefore, clorsulon detection is pivotal. In this study, density functional theory (DFT) and semiempirical metadynamics are used to investigate the properties of polypyrrole-based molecularly imprinted polymer (MIP-PPy) as a receptor targeting clorsulon detection. To address this aim, the electronic and physicochemical properties of PPy are studied, and the optimal imprinting conditions are calculated. The novelty of this study lies in the in silico characterization of the effects of PPy conformers on its electrochemical properties under different couplings. Next, the sensing mechanism via binding sites formed in the modeled imprinted polymer is described. The ratio of pyrrole monomers needed to form the optimal imprinted PPy for clorsulon drug detection is determined, and the solvent effect is evaluated. A water-based solvent was selected as the best medium and solvent for preparing the polymerization mixture, since it does not interfere with the optimized monomers and template molecules at a 16:1 ratio. Moreover, the αβ- and ββ-coupling present in PPy chains was considered and compared with the ideal configuration of all αα-coupled chains. Finally, the sensing mechanism and electrochemical properties of the in silico-designed MIP-PPy are discussed.

## Graphical abstract

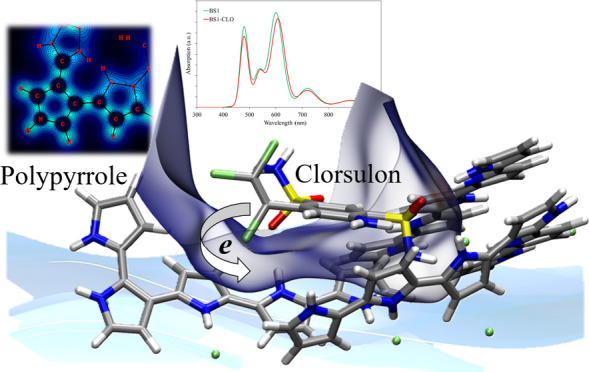

## Introduction

Clorsulon (CLO), 4-amino-6-(1,2,2-trichloroethenyl) benzene-1,3-disulfonamide, is widely used in veterinary medicine to treat liver flukes. However, medicament residues from treated livestock can enter the environment and pose risks to nontarget organisms.^1,2^ Such environmental dispersal poses a threat to both terrestrial and aquatic ecosystems, necessitating precise detection and enhanced monitoring.^3,4^ Studies indicate that the chronic exposure of humans to veterinary drug residues in food-producing animals is a rising public health concern.^5,6^ Traditional analytical techniques such as rapid ion-pair ultrahigh-performance liquid chromatography (IP-UHPLC),^7^ high-performance liquid chromatography-diode array detector (HPLC-DAD),^8^ mass spectrometry, and NMR^9^ offer accurate pathways for quantifying clorsulon. However, these methods are laboratory-bound and not suited for real-time environmental deployment. The current literature provides limited data on the onsite detection of clorsulon, despite the clear need for its monitoring. However, electrochemical and optical approaches show potential for in situ sensing due to their ease of use and rapid analysis.^10^

The evolution of sensor technologies, including molecularly imprinted polymer (MIP)-based sensors, represents a significant step toward improved public health monitoring through enhanced sensitivity and selectivity.^11–13^ MIPs are fabricated through a multistep process, starting with a prepolymerization step in which monomer-target analyte molecules form a complex in the selected solvent. Then polymerization is initiated to form a three-dimensional polymeric network. Following the polymerization, molecular imprints are created by removing the targeted analyte molecules, also known as the template. Consequently, the imprinted polymers possess tailor-made binding sites that are complementary in size and shape to the target molecule.

Density functional theory (DFT) has shown remarkable potential for studying the electrochemical properties of molecules in host–guest systems. To create models, atomic coordinates (e.g., created by a molecular editor or databases) are used, and the molecular system is then optimized at an appropriate level of theory to describe properties with the required accuracy. The efficient sampling of large or complex molecular spaces is a critical step in these models. Several enhanced sampling methods, such as replica-exchange, metadynamics, and umbrella sampling,^14,15^ are the most common ones at the atomistic level. However, high-accuracy enhanced sampling has been adapted to a low-cost quantum level to enable sampling of flexible molecules and large systems.^16,17^ The accuracy of calculated properties is expected once the sampling is rational and functional, and the basis set is adequate. Using DFT, noncovalent interactions (NCI) can be described, and the sensitivity of the receptors can be elucidated.^18–20^ The adsorption-based sensing mechanism of the target molecules has been studied and used to guide improvements in sensor performance.^21,22^ It is crucial to consider the conformers and rotamers if the molecules have a flexible structure.^23^ In the case of polymers, such as polypyrrole (PPy), different structures with distinct properties are formed during synthesis. Therefore, an imprinted polymer possesses different configurations. This influences the properties of the PPy polymer as the receptor, such as flexibility, chemical stability, and electrochemical activity. PPy may form different monomer bondings, namely, αα, αβ, and ββ coupling. Although the polymerization primarily proceeds via αα couplings to form linear conjugated chains, αβ and ββ couplings can also occur, resulting in branched and partially crosslinked structures. This branching is influenced by the polymerization conditions, such as the value of the applied potential during electropolymerization, current density, solvent, pH, and dopant anion.^24^ Doping of PPy is widely used in many applications to exploit its increased conductivity arising from extended conjugation.^25^ Several DFT and other computational studies have been applied to characterize the PPy polymer. Some notable previous studies focus on the effects of electron-donating and electron-withdrawing substituents,^26^ the doping process,^25,27^ the impact of the coupling mode on the doping ability of batteries,^28^ and the optical and electronic properties. Wasim et al.^29^ studied the sensing behavior of PPy toward nitrate ions using DFT modeling, and Aarab et al.^30^ identified PPy as an adsorbent for the removal of the pharmaceutical metronidazole from water using DFT modeling and confirmed the results using experimental data. The study by Zvirzdine et al.^31^ demonstrates the use of MIP-PPy for the electrochemical detection of salicylic acid (SA). The proposed electrochemical sensor with MIP exhibited a limit of detection (LOD) of 72 μM and a limit of quantification (LOQ) of 217 μM. The implemented DFT model showed the NCI between modeled bipyrroles and the analytes. The DFT model also revealed the nature of recognition, which caused the developed MIP to selectively detect SA. The study by Ankitha et al.^32^ developed an MIP-based electrochemical sensor for the detection of trimethylamine N-oxide. DFT in this study was used to identify 4-amino phenyl benzoic acid as the best functional monomer for sensor development due to its high sensor response and a high, negative binding energy. Xie et al.^33^ designed an MIP-based sensor for morphine. This study was supplemented with computational insights. The analyses of molecular electrostatic potential (MEP) and orbitals interaction region indicator (IRI) were visualized, and the identified interaction types assisted in finding optimal prepolymerization. The workflow of this study to calculate the electronic and interaction properties of the molecular system included a conformational search to find the optimal configuration, then semiempirical GFN2-xTB, and later DFT. The experiment confirmed the optimal synthesis system. Rebelo et al.^34^ developed a disposable electrochemical MIP sensor for atorvastatin. To this aim, DFT calculations were carried out to find the optimal monomer based on the binding energies of screened monomers toward the target. Then, the molecular dynamics (MD) simulation was performed on the prepolymerization mixture to study interactions via radial distribution functions (RDFs) and hydrogen bonding analyses. In another study,^35^ the authors optimized an MIP synthesis condition for furazolidone detection by a similar strategy. The study by Adeleke et al.^34,36^ demonstrates the development of an epinephrine-imprinted acrylic-acid-based MIP. DFT and MD simulations were performed to determine optimal prepolymerization conditions, including binding energies and thermochemistry parameters such as miscibility and Flory–Huggins’ factors at varying temperatures. The obtained optimal mixture was further analyzed as an MIP with specific volume, density, mean-square displacement, intensity, equilibrium energies, and radius of gyration analyses at different temperatures. Rajaee et al.^37^ performed DFT-based development of an MIP for renal failure biomarkers by optimizing prepolymerization mixtures and studying the selectivity of pyrrole (Py)- and acrylamide-based MIPs. In this study, the complex formed by the optimized prepolymerization mixture was considered the imprinted region, and binding scores were analyzed as a measure of selectivity.

To the best of our knowledge, at the time of this work, no MIP has been developed for clorsulon detection. Therefore, this study aims to provide a full theoretical approach to study the MIP-PPy for clorsulon sensing. The significance of this study lies in evaluating the clorsulon detection mechanism on the imprinted PPy. In this article, the optimal template-to-monomer ratio and solvent effect are assessed. Conformer and rotamer studies are included to model more realistic oligopyrroles, and the electrochemical characterization of PPy is presented. Later, the imprinting efficiency and the PPy imprinting process are evaluated by comparing with nonimprinted PPy (NIP). The novelty of this work elucidates the imprinting process, recognition, and sensing mechanism of clorsulon by modeling specific binding sites and comparing with nonspecific interactions. The key idea is to demonstrate finite molecular models of the imprinting PPy under in silico optimized prepolymerization conditions to investigate the size and density of binding sites and their correlation with charge-transfer values in these imprinted regions, and binding energies toward the analytes. The workflow of the employed calculations is shown in Figure S1.

## Results and Discussion

### Optimization of the Monomer-to-Template Molar Ratio

Finding the optimal monomer-to-template stoichiometry is essential for the synthesis of an imprinted polymer as a receptor with maximally efficient binding sites for target analytes. Insufficient monomer coverage fails to establish a firm, complementary binding pocket around the template, whereas excess monomers risk encapsulating the analyte within the polymeric matrix, rendering it inaccessible.^38^ The increasing number of Py monomer molecules (from 1 to 18) around one clorsulon template molecule resulted in complexes with different ratios, ranging from 1 to 18. Three complex sets were built and optimized, and the average of binding energies was calculated, as it is shown in Figure 1A. The sampling was to place the template at the geometrical center, maximizing interactions with monomers. The binding energies of Py monomers around the template were calculated using eq 1. Once the multicomponent complexes are created, the binding energies are contributed by all molecules. Here, monomers and the clorsulon template formed complexes with different monomer-to-template ratios. Therefore, the average of binding contribution among monomers and between the monomers and the template was gathered and compared along with the binding energies of the entire complexes. The total binding energy of the entire complex consists of the sum of the binding energies of all molecules. The addition of monomers gradually reduced the share of monomer–template binding energies of the total, and an incremental increase in interactions among monomers occurred. An excessive number of monomers can reduce the imprinting efficiency by permanently trapping the template, making it impossible to remove later. Therefore, based on the binding energy, the 16:1 ratio for Py–clorsulon was selected as the optimal one. The addition of more monomers did not increase the binding energy of the monomer–template complex noticeably but increased the pace of interactions and affinity among monomers.

**Figure 1:**
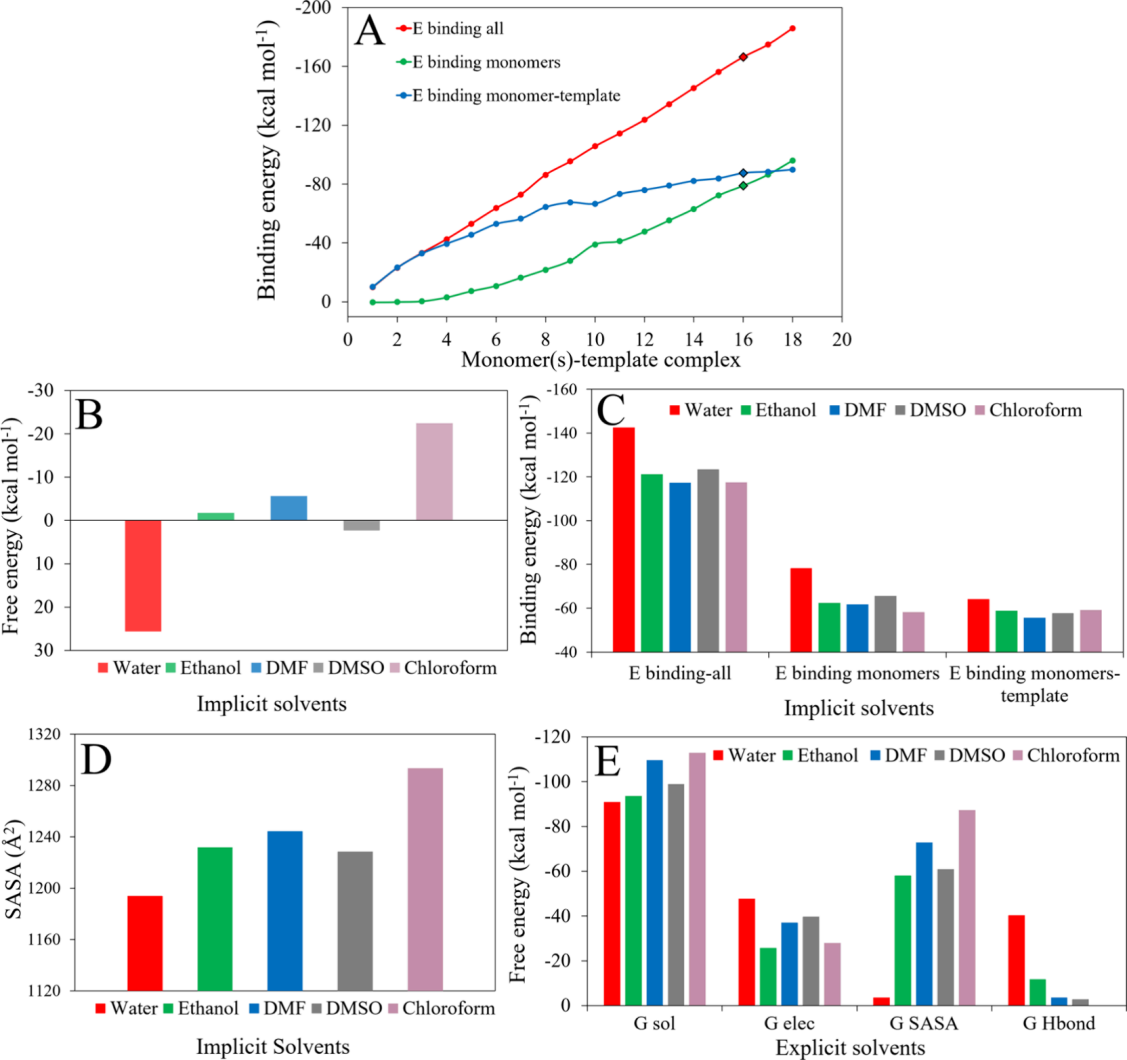
(A) Comparison of changes in the binding energies among Py monomers, between monomers and the clorsulon template, and the entire complex. (B) Free energy of implicit solvation of the optimized 16:1 ratio of Py to clorsulon complex. (C) Effect of the solvation on binding energies of the prepolymerization complex. (D) SASA of implicit solvents. (E) Explicit solvation of the complex and calculated free energies.

### Assessment of the Solvation Effect

The prepolymerization mixture contains monomers and an analyte molecule in a solvent system. The choice of solvent plays a crucial role in the interactions between monomers and the analyte, which acts as the template. It should be noted that all calculations for the solvation effect were implemented on a complex of Py–clorsulon with a 16:1 ratio, as described in the previous section. For explicit solvation, 10 solvent molecules were added. To understand the effect of the solvent on the complexes, common solvents were considered. Water, ethanol, DMF, DMSO, and chloroform have dielectric constants of 78.4, 24.9, 37.2, 46.8, and 4.7, respectively. The selection provided solvents with varying polarity values, protic or aprotic, and hydrophobic or hydrophilic properties. The SMD model comprises two components calculated by eq 2. The bulk electrostatics are described by the polarizable continuum model (PCM) and the cavity-dispersion solvent-structure (CDS) model is used for nonelectrostatic contributions. The latter accounts for short-range interactions between solvent and solute. This contribution relies on terms related to the geometry-dependent proportionality constants, so-called surface tensions of each presented atom, with respect to the solvent-accessible surface area (SASA). However, in explicit solvation, the Gibbs free energy is calculated using eq 3 as the sum of polar and correction for nonpolar plus hydrogen bonding and the shift constant contribution. The free energy of solvation by polarity is calculated in the electrostatic-potential-related term *G*_elec_. The *G*_SASA_ term represents the computation of the free energy required to form the solute–solvent cavity and to consider the solute–solvent dispersion interactions and a correction for H-bonds formed between the solvent and solute, denoted as *G*_H‑bond_.

Figure 1B represents the free energy of the SMD implicit model solvation for the 16:1 Py-clorsulon complex. The positive values of 25.61 and 2.31 kcal mol^–1^ were obtained for water and DMSO for the solvation of the entire complex, respectively. The most favorable was chloroform due to its nonpolar nature with −22.40 kcal mol^–1^. DMF and ethanol showed negative free energies of −5.63 and −1.70 kcal mol^–1^, respectively.

Figure 1C shows the binding energies of complex components: *E* binding all, *E* binding monomers, and *E* binding monomer–template in the presence of the solvents. The highest energies were obtained for the water solvation. The lowest binding energy of the monomer–template was in DMF (−55.68 kcal mol^–1^), followed by DMSO (−57.83 kcal mol^–1^). However, complexes in water showed considerably higher binding energies than those in other solvents. Figure 1D displays the SASA (Å^2^) values obtained from the complex in the SMD implicit solvent. The lowest and the highest SASA values for water and chloroform stem from the polar and nonpolar properties of the solvents. There is an inverse correlation between SASA and the binding energies of the entire complex. The correlation exposes the solvent’s ability to form solute–solvent cavities and short-range interactions. The higher solute–solvent cavitation reduces the binding affinities by interfering with the nonpolar hydrophobic interaction among complex components.

Figure 1E demonstrates the free energy values for the complex obtained after adding 10 explicit solvents. The values reveal the portion of each term in the total. *G*_sol_ explains more solvent effects, even though only a limited number of solvent molecules were placed. Moreover, the results are in good agreement with the SMD solvation model. The least negative *G*_sol_ was for water, and the most for chloroform. The lowest SASA value for water as the solvent corresponds to the obtained free energy due to the highest polarity of water and, consequently, the least short-range nonpolar interaction with the solute. High values of *G*_H‑bond_ and *G*_elec_ indicate the relative immiscibility of molecules with polar interactions. Higher *G*_H‑bonds_ were found in ethanol, followed by water, as two protic solvents compared to polar aprotic DMF and DMSO. Chloroform as the solvent exhibited the largest value of *G*_SASA,_ which explains the main reason for the most favorable free energy of solvation among all of the applied solvents. This can be interpreted as the significance of H-bonds, electrostatic polar, and nonpolar interactions between the solute and the solvent. However, in comparison, the contribution of nonpolar hydrophobic interactions is predominant, minimizing water–solvent–solute interactions due to the lowest solvent cavitation. Therefore, higher interactions among monomers and between the monomer and the template were observed. Chloroform, a nonpolar solvent, showed the highest SASA and most favorable Gibbs free energy and the highest short-range interactions. DMF followed by chloroform showed the second most favorable *G*_sol_, with relatively higher electrostatics but lower SASA and corresponding *G*_SASA_.

Overall, the interfering effect of water solvation was found to be minimal with the lowest short-range interactions. This boosts the formation of binding sites with more specific geometry for the target analyte.^19,39^

The result of the solvation effect can be utilized in the extraction process, although it is not covered in this work. However, the lowest binding energy between the monomers and template found in the DMF solvation and the second favorable *G*_sol_ for explicit solvation shows promise to be used for the removal of clorsulon from the synthesized polymer. The free energies of solvation from the implicit SMD model for isolated clorsulon were 5.43, 2.58, −2.72, −1.47, and −1.21 kcal mol^–1^ for the listed solvents, respectively. This emphasizes the suitability of DMF with the highest potential to disturb interactions and remove the analyte from the synthesized polymeric binding site.

### Conformer and Rotamer Analysis

Conformational analysis is essential in host–guest studies, as molecules typically exhibit multiple accessible local minima.^40^ Therefore, using a structure containing a single molecule statically often does not describe the binding geometry and may introduce misinterpretation in the predicted binding energies. The conformational changes, particularly in flexible molecules, cause penalties upon binding. Thus, conformation and rotation must be explicitly sampled.^41^ Therefore, robust geometry optimizations with conformational searches at lower levels of theory are required to adequately explore the phase space.

#### Template Characterization

The geometries of clorsulon conformers were optimized in the gas phase (vacuum) and an implicit water model. The static optimization focused on low-energy structures arising from rotatable sulfonamide functional groups given in the PubChem data set. The relative electronic energies of the conformers with reference to the main conformer revealed that all conformers are within 1.0 kcal mol^–1^. The water solvent altered their relative energy, and the poor solubility of clorsulon in water was evident from *G*_sol_ values. Such small energy differences imply that multiple conformers are thermally accessible and significantly populated at room temperature. The implemented ab initio molecular dynamics (AIMD) (Figure S2) shows the feasibility of conformational change within a short period of 1500 fs in a water medium at room temperature. The rotation of the disulfonamide functional group makes the molecule accessible to different conformers for adsorption and binding to an adsorbent. The analysis emphasized the individual low-energy conformers to illustrate the flexibility of the disulfonamide moiety, which enables the molecule to adopt different spatial arrangements that are potentially favorable for adsorption and binding to a specific tailor-made adsorbent surface, such as MIPs. Four conformers of clorsulon are shown with the electrostatic potential map (MEP) in Table 1.

**Table 1:**
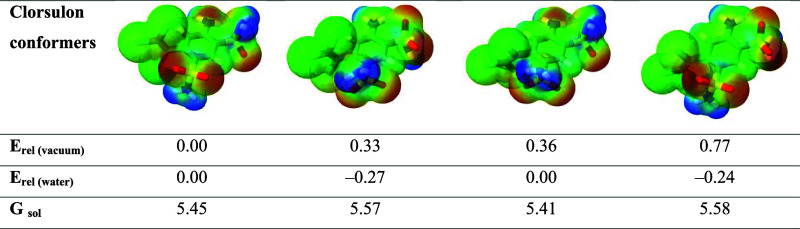
MEP Map of Clorsulon Conformers and the Relative Electronic and Solvation Energies in Vacuum and Water in kcal mol^–1^

The conformers depicted the MEP of the molecules, where functional groups created regions with electron access (red) and deficiency (blue). The oxygen and nitrogen atoms are electron-rich because they pull electrons. Therefore, hydrogen atoms of amines have more electron deficiency due to the adjacent electronegative atoms than other hydrogen atoms in the ring. The small relative energies in the host–guest system led to fluctuations and altered binding affinities. Therefore, a study of conformer changes in the medium is required. Likewise, all four conformers were considered similarly in the next steps for imprinting sampling.

#### Polymer Characterization

Three configurations of PPy were used for conformer/rotamer analysis, including oxidized (bipolaron 2+ charged singlet close-shell, and triplet open-shell) and reduced states of PPy. For each state, αα, αβ, and ββ coupling was modeled to study the topology and electrochemical properties of each chain. To achieve this aim, an ideal PPy model chain that consists of all αα coupling and two similar PPy models, one with one αβ coupling and another with one ββ coupling, were evaluated. This made it feasible to study the effect of different couplings more realistically once αα is dominant.

Triplet and singlet states of bipolaron PPy chains in three different configurations are compared with the corresponding reduced state to analyze the effect of chain length in αα, αβ, and ββ coupling. The chain growth was modeled on one side of the αβ and ββ, where dimers were incrementally added. Therefore, oligomers containing 6, 8, 10, 12, and 14 units in length were modeled. The conformers of each in different states were obtained. The average energies of the bipolaron PPy chains were compared with the corresponding reduced state. The relative energies, presented in Figure 2A, showed higher energies for the triplet state of bipolaron in all chain lengths and configurations. The analysis revealed that the relative energies of conformers are *E*_αα_ < *E*_αβ_ < *E*_ββ_ in the singlet state and *E*_αα_ < *E*_ββ_ < *E*_αβ_ in the triplet states. Also, in the 6-mers of PPy in triplet states, *E*_αα_ > *E*_αβ_ > *E*_ββ_ is observed, showing the lowest relative energies for ββ. The reduction in relative energies with the chains growing showed that the difference in the energies of the triplet and singlet states becomes smaller. Overall, the singlet state has lower energies and is favorable in the electronic configuration in the bipolaron state of PPy. Figure 2B demonstrates the MEP of bipolaron PPy chains with an ideally coupled structure (I), one bond αβ (II), and one bond ββ coupled (III). The electron path is observable where regions are red and yellow, showing electron accessibility along the chains. The electron deficiency regions (blue-green) are visible on the edges around hydrogens.

**Figure 2:**
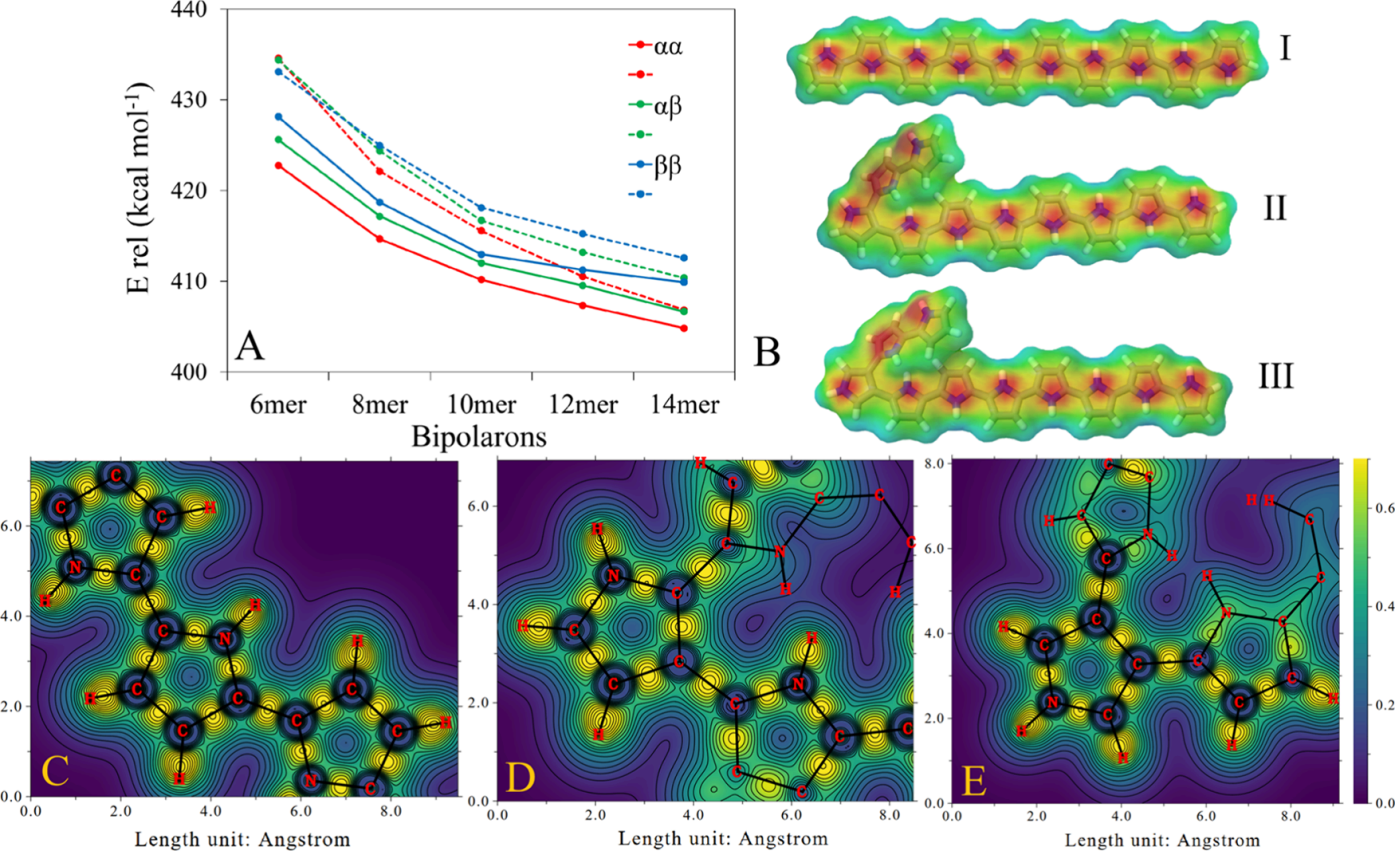
(A) Relative energies of bipolaron states compared with reduced states of PPy. The solid lines are singlet and dashed lines are triplet states of PPy. (B) MEP of the singlet state of bipolaron PPy, (I) with all bonds in αα, (II) one bond αβ, and (III) one bond ββ coupled. (C–E) Topology analysis of singlet-state bipolaron PPy by the LOL with all bonds αα, one bond αβ, and one bond ββ coupled, respectively.

The localized orbital locator (LOL) (Figure 2C–E) represents the topology of PPy chains in plane where different couplings occurred. The LOL shows that all αα couplings (Figure 2C) form a planar and rigid structure with orbitals localized, which provides a smoother pathway to the electrons along the chain. However, in Figure 2D,E, the atoms and orbitals appeared out of plane and appeared faded. The folded chains made distorted paths, which are observable in the PPy with the presence of αβ and ββ, respectively.

Table 2 includes two parts: one is for all conformers derived from Crest software and the other is for the electrochemical analysis of the best obtained conformer with the higher level (r2SCAN0/def2-mTZVPP). The structures’ end-to-end distances were measured to evaluate the polymer folding and flexibility in different coupling types. Further calculations were dedicated to the singlet state as the more energy-favorable of the bipolaron PPy. In total, six configurations were used for conformer/rotamer analysis, including oxidized (bipolaron singlet close-shell, 2+ charged) and reduced states of PPy that consist of 10 Py monomers. In the case of the PPy chain with one αβ or ββ coupling, the induced *cis* position was observed in the conformers. It was observed that the αβ coupling and the ββ coupling fold the chain. The analysis of conformers/rotamers revealed that the αβ coupling and ββ coupling induce the *cis* conformation among adjacent monomers due to angle torsion and bond relaxation. Overall, the *cis* position in the middle of the chain had lower energy, while the ends of the chain had higher energy. Additionally, two separate *cis* positions were more energetically feasible when they were in a trans position to each other. Three adjacent linked Py monomers in the *cis* position were observed at higher energies than two separate cases of *cis* positions. Similarly, three *cis* monomers were observed to be more stable in the middle while having the higher energy when shifted more toward the chain ends. The average end-to-end distances and the corresponding standard deviation showed a considerable reduction in the chains’ length due to the folding in the αβ and ββ couplings (Table 3).

**Table 2:** Average End-to-End Distance (Å), Its Relevant Standard Deviation (SD), and the Percentage of the *cis* Position in the NH–NH of PPy Conformers with All Bonds αα, One Bond αβ, and One Bond ββ Coupled^a^

	all conformers			best conformers							
polymer	end-to-end (Å)	SD	%*cis*	BLA (Å)	IP (eV)	EA (eV)	*E*_g_ (eV)	μ (eV)	η (eV)	ω (eV)	Δ*N*_max_ (*e*)
αα-rePPy	28.67	4.49	45.2	0.042	4.45	0.86	3.59	–2.65	1.79	1.96	1.48
αα-oxPPy	28.84	5.05	42.3	0.027	4.81	3.91	0.90	–4.36	0.45	21.08	9.67
αβ-rePPy	20.03	6.01	50.6	0.044	4.50	0.82	3.68	–2.66	1.84	1.92	1.44
αβ-oxPPy	21.73	6.17	43.5	0.035	4.97	3.91	1.07	–4.44	0.53	18.49	8.33
ββ-rePPy	20.13	6.08	48.4	0.047	4.52	0.79	3.73	–2.66	1.86	1.89	1.43
ββ-oxPPy	19.98	6.96	49.4	0.037	5.06	3.91	1.16	–4.48	0.58	17.38	7.75

a The electrochemical properties of the best conformer of PPy in the bipolaron singlet state as the oxidized state (oxPPy) and the reduced (rePPy) state (eV).

**Table 3:** Hirshfeld Surface Analysis between the Template and Binding Sites Exposed the Surface Area of Atoms in Contact, the Created Imprinted Volume, and the Complex’s Density

complex	surface (Å^2^)	volume (Å^3^)	density (g/cm^3^)
BS1-CLO1	322.5	817.5	3.33
BS2-CLO2	359.4	665.5	4.09
BS3-CLO3	351.8	652.2	4.18
BS4-CLO4	316.7	873.0	3.12
BS5-CLO1	344.5	747.0	3.65
BS6-CLO2	292.5	887.4	3.07
BS7-CLO3	298.13	817.5	3.33
BS8-CLO4	245.56	1062.3	2.56

Bond length alternation (BLA) was calculated using eq 4 to evaluate the PPy in different couplings and the oxidation effect on the conjugation. The ionization potential (IP) and electron affinity (EA) were approximated from HOMO and LUMO energies. The chemical potential (μ) and hardness (η) were derived from eqs 5 and 6, respectively. Then, the global electrophilicity (ω) index and the maximal charge transfer (Δ*N*_MAX_) were calculated using eqs 7 and 8, respectively. PPy showed higher EA in oxidized bipolaron, which is due to the loss of the electron. A slightly higher IP is observed in oxidized cases. Therefore, much smaller band gaps are presented for the oxidized PPy. The measure of BLA showed smaller bond alteration in the oxidized state, revealing extended conjugation and doping of PPy. However, the presence of different couplings reduced the conjugated system and larger values of BLA were detected. This increases the BLA and consequently reduces Δ*N*_max_ and other relevant parameters, showing the adverse impact of different couplings on the conjugation of PPy (Table 3).

The application of the conducting polymer—PPy—as the receptor to recognize the target molecules via imprinted regions requires a greater degree of freedom in the polymeric backbone. The rigid polymer with all bonds in αα coupling has smoother pathways for electron movement. However, the imprinting of small molecules relies on the receptor’s flexibility to create complementary binding sites. Therefore, folded chains, despite their undesired effects on conjugation, offer more freedom for the chains to move to form imprinted areas.

### Imprinting of PPy with the Clorsulon Template

Once the polymerization takes place in the presence of the analyte molecules as the template, a polymeric network is fabricated around the analyte. This process was mimicked by performing geometry optimization of the assembled PPy oligomers around the clorsulon. The oligomer–template complexes were modeled regarding the obtained optimal ratio and solvent. In this model, we included two PPy pentamers and one PPy hexamer, reflecting the part of the polymeric network that interacts with the analyte molecule and forms cavity-binding sites. The models have a limitation in their chain length effect on the structural parameters of PPy, such as entanglements among chains and interactions with multiple target analytes. The oligopyrroles contain *cis* positions and different couplings, as the nonideal model that occurs in the chain during propagation. This modeling was considered to better represent the structural heterogeneity present in MIP-PPy. In this step, a conformer and rotamer search was performed, and the top three structures were selected to form different complexes, aiming to model eight binding sites (BSs), two for each clorsulon conformer. The complexes were created first by finding the conformers of oligomers in the oxidized state. Then the three best conformers were used to make eight complex sets of PPy with four conformers of clorsulon. Chlorine counterions (Cl^–^) were added to the complex to neutralize the system, reflecting the diffusion of counterions into the bipolaron, a positively charged polymeric network.

A Hirshfeld surface analysis was performed to analyze the imprinted surfaces, which showed the size and shape of the cavities as the tailor-made binding sites for clorsulon. Figure 3 shows the obtained surfaces sampled between clorsulon and the binding sites derived from eq 9. The dominant pairwise elements in contact between fragments were H–H, O–H, and Cl–H in all complexes. The oxygen atoms from clorsulon play a key role in complex stabilization and NCI interactions.

**Figure 3:**
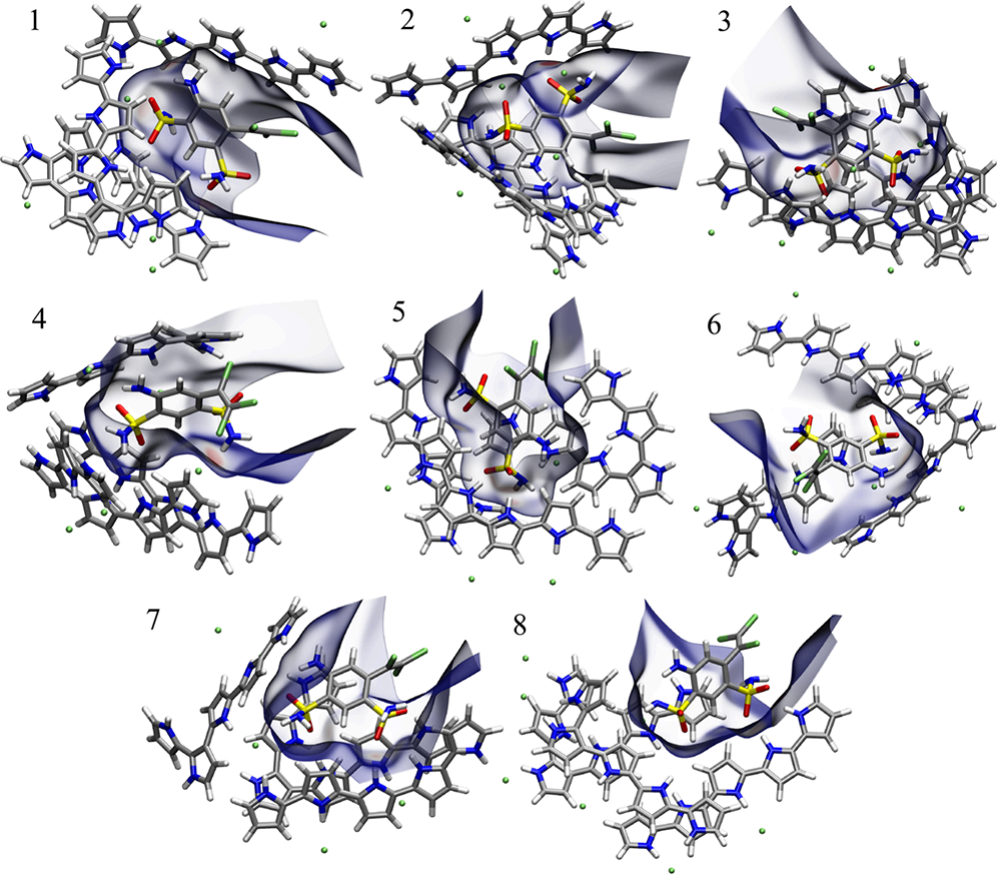
Graphical representation of BS1–8 imprinted with clorsulon conformers. BS1 and BS5 imprinted with conformer 1, BS2 and BS6 with clorsulon conformer 2, BS3 and BS7 with clorsulon conformer 3, and BS4 and BS8 with clorsulon conformer 4. The Hirshfeld surface was sampled between the template and the binding sites.

Hirshfeld surface analyses reveal the surface area (Å^2^), volume (Å^3^), and density (g/cm^3^) of the rebound clorsulon molecules within the binding sites. The values reported in Table 3 correspond to the graphical isosurface shown in Figure 3. The analysis shows cavities have different dimensions and densities. BS8 exhibits a loose binding site with the smallest density, where clorsulon is partially imprinted in PPy. However, BS2 and BS3 are tightly complexed and demonstrate the highest densities, the largest surface, and the smallest volume. In comparison, other complexes show values in between those of the majority of the complexes.

The analysis showed that relatively different imprinted regions were created in terms of size, shape, and corresponding density. The comparison shows that binding sites with a density greater than 3 g/cm^3^ are the majority. However, the result exposed substantial differences in the dimensions of the most and least dense complexes. An indication of the need for a careful extraction and rebinding process to achieve the highest possible accuracy.

#### NCI Analysis

NCI analysis is well-suited for the identification and visualization of NCIs derived from eq 10. In this study, NCI analysis was performed using Multiwfn software with high-quality grid for the qualitative study of inter- and intramolecular interactions.

It was found that hydrophobic interactions were the dominant in the formation of the complex. Figure 4A shows π-sulfur (yellow), electrostatic attractive charge and π­(pi)–anion interactions (red), hydrophobic stacked and T-shaped π–π (purple) and π–alkyl (green) interactions, while Figure 4B shows hydrogen and halogen bonds (depicted in blue dashed lines). The counterions formed two types of strong interactions: one is the strong halogen ion-dipole bonding with the amine functional group and another is π–anion interactions with the chains having electron deficiency. Figure 4B shows the reduced density gradient (RDG) isosurface, which indicates the interaction regions. The RDG isosurface of all binding sites is presented in Figure S3. Attractive halogen and H-bonds have negative values: sign­(λ_2_)­ρ, where ρ has a positive value and λ_2_ has a negative value. The H-bond spikes are shown in the blue-green (which are weaker) and blue regions (which are stronger). The π-donor H-bond can be observed in the complexes with a smaller attraction force as appearing in the green isosurface. The Cl^–^ anion interactions with PPy and clorsulon have been shown to be the strongest. van der Waals (VDW) interactions, such as dipole–dipole, dipole-induced dipole, and London dispersion interactions, are shown in light green. These interactions have both λ_2_ and ρ with small values close to zero. The dominance of the polar interactions arises from the extensive hydrophobic π–π and H-bond π-donor electrostatic interactions. Red spikes in the positive region of sign­(λ_2_)­ρ represent repulsive forces, mainly stemming from steric hindrance at the center of the rings and intramolecular functional groups.

**Figure 4:**
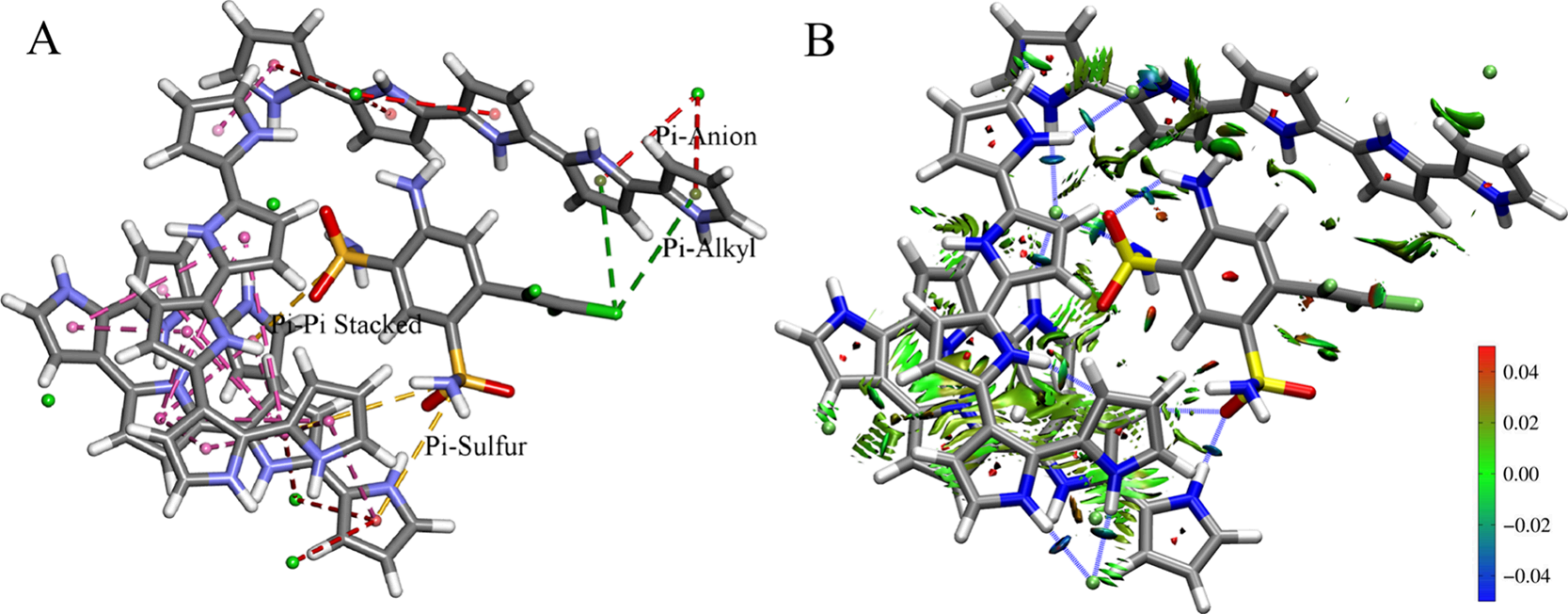
NCI analysis of the clorsulon molecule inside BS1-CLO1: (A) hydrophobic interaction and (B) RDG isosurface; dashed lines represent hydrogen and halogen bonds.

### Assessment of Sensitivity

#### Charge Decomposition Analysis

Figure 5A,B represent the results of the charge decomposition analysis (CDA) of the binding site–clorsulon (BS1-CLO1) complexes. CDA was employed to understand the mechanism of sensing the target molecule. CDA was used to delve into the underlying details of charge transfer, where electron donation (d) and back-donation (b) between two fragments are due to the contributed MOs. Therefore, electron transfer is composed of contributing MOs. The number of electrons involved in repulsive polarization shown in Figure 5B also indicates the repulsive Pauli exclusion due to the orbital overlap. In all complexes, electron back-donation (b) was greater and donation from binding sites to clorsulon molecules was smaller. Therefore, electron net transfer and electron polarization effects are carried from clorsulon to PPy (b > d) due to the electron deficiency of bipolaronic PPy chains. The electron transfer occurs via lone pairs of oxygen in the clorsulon guest. Guest-induced carrier modulation was observed when clorsulon acted as an electron donor to the PPy-based binding site. This reduced the net positive charges of bipolaronic PPy. The value of d–b highly depends on the clorsulon template interactions with Cl^–^ counterions. Stronger NCI interactions between PPy and clorsulon occur in denser systems, resulting in higher electron transfer in both directions. This is due to the accessibility of Cl ions due to the clorsulon molecule being more in denser complexes, alongside the random vicinity of the contributing atoms. Therefore, lower values of d–b were observed when the Hirshfeld surface area of atom pairs showed a higher surface area of clorsulon atoms with Cl^–^ counterions. The NCI analysis via RDG isosurface revealed an extended green isosurface, indicating the interaction of clorsulon with the electron-deficient π-conjugated system of PPy. This brings about multiple molecular orbitals (MOs) interacting and overlapping and causes charge delocalization in the imprinted region.

**Figure 5:**
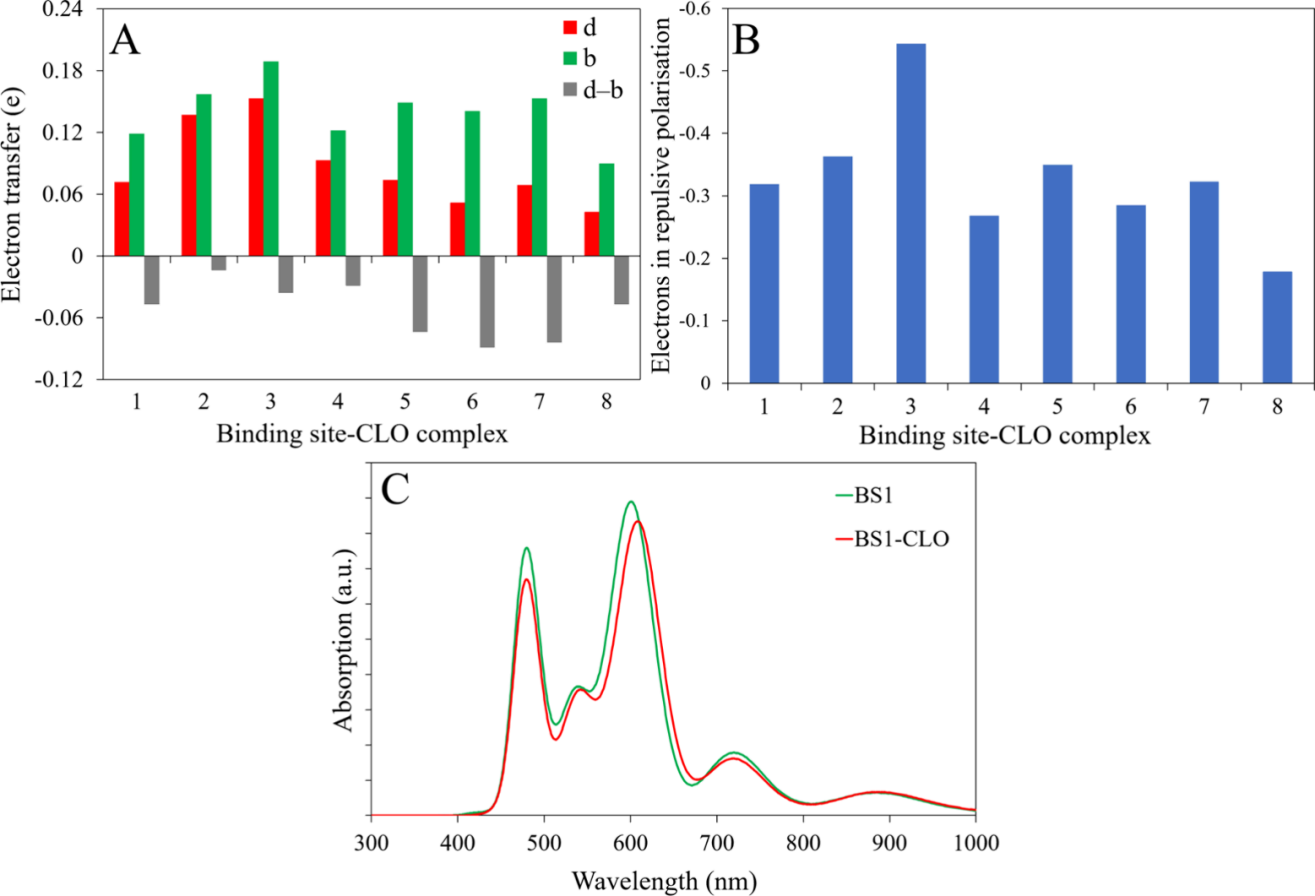
(A) CDA of BS1–8 imprinted with clorsulon (CLO) conformers. Electron donation (d) from the binding site fragment and electron back-donation (b) from clorsulon to the binding sites occur. (B) Repulsive polarization represents the number of electrons involved in the repulsive forces among MOs. (C) Theoretical UV–vis spectrum of BS1 and its complex with the clorsulon template molecule in the C-PCM water model.

#### UV–Vis Spectrum Analysis

The UV–vis spectrum (C) of the BS1-CLO1 complex is displayed in Figure 5C. Intragap absorptions arise from electronic transitions having in-gap states formed upon doping. Therefore, polaron and bipolaron-doped molecules undergo sub-band transitions.^42^ As a result, additional low-energy absorptions appear in the spectrum. Conjugated and doped structures dissolved in the polar solvent (water) exhibit bathochromic (red) shift in the peaks where λ_max_ emerges in the higher wavelength. This is due to enhanced delocalization and stabilization of the charged PPy in the polar medium. The peak at 479 nm (2.59 eV) is attributed to interband π–π* transitions, characteristic of neutral PPy.^43^ The weaker adsorption peak at 542 nm (2.29 eV) corresponds to polaronic transitions. The bipolaron band transition shows an absorption peak at 600 nm. The bathochromic (red) shift of this peak from 600 to 608 nm (respectively 2.07 to 2.04 eV) is from the presence of the clorsulon guest inside the cavity with hydrophobic interactions in a water polar continuum medium.

Back-donation via lower lying MOs partially quenches bipolaron states, which indicates the hypochromic effect in peaks. Fewer charge carriers cause lower absorption intensity. The clorsulon molecule stabilizes oxidation states by lowering the overall transition energy relative to the pure bipolaron band. The broader peaks of the visible near-infrared (NIR) absorption at λ_max_ 719 nm (1.72 eV) and 888 nm (1.40 eV) can be assigned to the subgap transitions of bipolaronic PPy chains.

The CDA revealed that the presence of clorsulon as the guest molecule induced electron transfer from the clorsulon molecules to the binding sites, thereby reducing the doping effect of the bipolaronic PPy. However, exposure of Cl^–^ counterions to clorsulon increased back-donation and reduced the net transfer (d–b). The UV–vis spectrum of BS1 and its complex with the clorsulon template as the guest showed the doped structure of PPy with absorptions from subgaps caused by heavy oxidation. The effect of the presence of clorsulon in the binding site also demonstrated good agreement with the CDA.

#### DOS Analysis

The center of the total density of state (TDOS) for the complex was found to be −6.36 eV, the center of the partial DOS (PDOS) for the PPy fragment was −6.53 eV, the center of PDOS was −4.26 eV for Cl^–^ ions and −7.05 eV for the clorsulon molecules. The vertical dashed lines (−5.44 and −4.52 eV) represent the HOMO level of the complex. The overall doping effect of Cl^–^ ions and the role of the extended conjugate PPy are observable in the DOS curves where the corresponding line crosses the dashed line of HOMO. The sub-band of heavy oxidized PPy chains is present at 4.08 eV in Figure 6A, and it vanishes in the reduced PPy, as shown in Figure 6B. The presence of clorsulon shifted the TDOS to lower energies, farther from the HOMO level. The relative height of curves can provide insights into MO compositions. DOS shows high sensitivity of the doped imprinted PPy to the presence of clorsulon molecules, despite the fact that the clorsulon frontier molecular orbitals (FMOs) exhibit lower energies. Substantially larger π MOs of PPy are noticeable in all of the low-lying and conduction bands.

**Figure 6:**
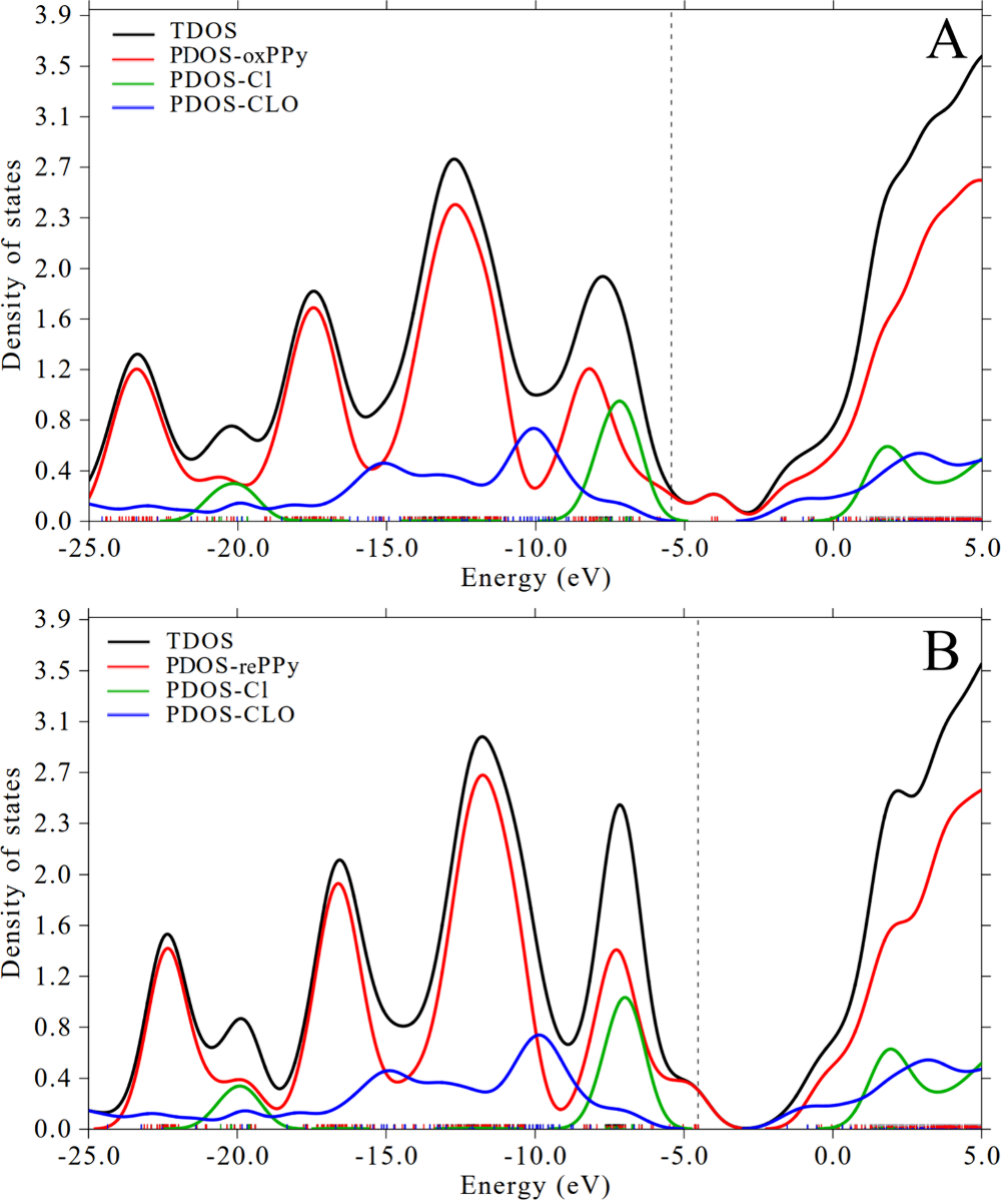
DOS of the total first BS1-CLO1 complex and each component, including PPy, Cl^1–^ counterions, and clorsulon (CLO) template molecule: (A) bipolaron PPy as the oxidized state of BS1 and (B) reduced PPy of BS1.

MO interaction diagrams shown in Figure S4 represent the orbitals contributed in each fragment (fragment 1 is BS1 and fragment 2 is the clorsulon template) and their energy and contribution to the complex in the oxidized bipolaron and reduced states, respectively. No contribution from clorsulon is observed, while doped PPy has shown a substantially reduced energy in the LUMO. However, in the reduced state, LUMO is contributed from clorsulon orbitals. Overall, complexation of the binding site with clorsulon affects orbital energies with lower lying orbitals more dominantly and has a minor effect on the band gap in the oxidized state.

Spectral and electronic characteristics of PPy complexed with clorsulon showed that clorsulon back-donates an electron and reduces the charges of the PPy bipolaron. The sensing mechanism can be predicted through the reduction of charge carriers and an increase in resistance, as well as a hypochromic effect in the UV–vis spectrum.

### Assessment of Selectivity

#### Binding Energy

Binding energies were calculated by eq 1 and are shown in Figure 7A. The goal was to determine the binding affinities of the binding sites toward the clorsulon template (380.7 g/mol) and analyze the effect of conformer changes. Amoxicillin (AMX; 365.4 g/mol) drug or SA (137.11 g/mol) was introduced into the binding sites randomly to evaluate the selectivity as interfering molecules. The large difference of binding energies with binding sites represents the varied affinities, ranging from −18.62 to −41.21 kcal mol^–1^, and subsequently different potential recovery times. However, high binding energies are noticeable in all binding sites for clorsulon (28 atoms, C_8_H_8_C_l3_N_3_O_4_S_2_). The introduced AMX was considered as deprotonated AMX (43 atoms, C_16_H_18_N_3_O_5_S^–^; p*K*_a_ 2.6), with three conformers, as shown in Figure S5, in BS1, 3, 4, and 6. This resulted in lower binding affinities for AMX in BS1 and 3 but higher affinities in BS4 and 6. SA was also considered in the deprotonated state (salicylate ion, 15 atoms, C_7_H_5_O_3_^–^; p*K*_a_ 2.78), and its comparable small size gave higher degree of freedom values to move and find the best position inside BS2, 5, 7, and 8. In all cases, lower binding energies for the conformer-changed clorsulon are noticeable in comparison to clorsulon acting as the template for its specific binding site. Thus, one challenge is conformer changes, which drastically reduce the binding energies for clorsulon when another conformer of clorsulon is placed in the binding site created by a different conformer. This is due to the conversion of attractive forces into repulsion in the regions where the new conformer poses a new geometry and introduces new electron donors or acceptors. The MEP of clorsulon conformers in Table 1 show the rotation of disulfonamide functional groups with the electrostatic potentials.

**Figure 7:**
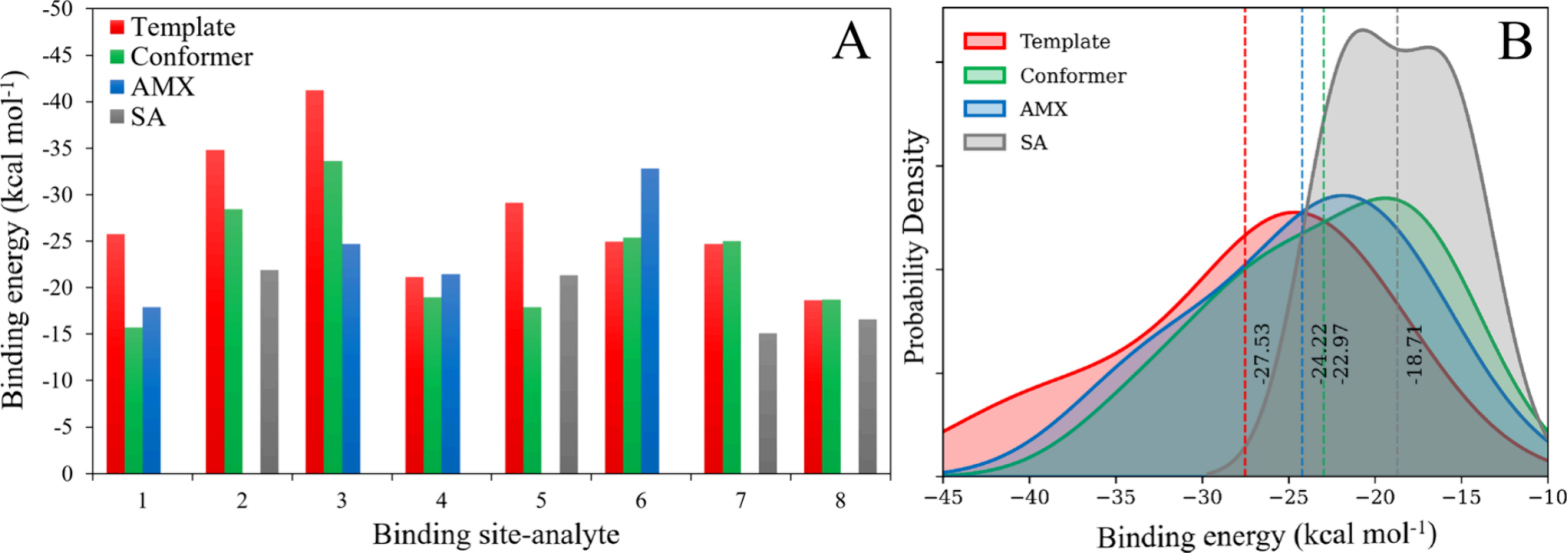
(A) Binding energies of the clorsulon template and its conformers as analytes and deprotonated AMX and SA as interfering molecules in the binding sites. (B) KDE curves of complexes show the probability density of binding energies for the molecules.

The binding energies of the studied analytes were further analyzed using kernel density estimation (KDE) curves shown in Figure 7B. The KDE curves provide a smoothed representation of the probability density of the binding energy values, highlighting the overall shape of the distribution and the central tendency of each molecule. The average of binding energy values also expresses the highest affinity toward the template. The template shows the most favorable (−27.53 kcal mol^–1^) binding energy, with its distribution shifted toward lower energy values compared to its conformer, AMX, and SA. However, the change in conformer placed the average binding site at −22.97 kcal mol^–1^ and shifted the curve by two positive values. The narrow curve for SA is obvious compared to others due to its smaller size and correspondingly lowest flexibility. On the other hand, AMX has a noticeably large average binding energy of −24.22 kcal mol^–1^ and a wide distribution stemming from its larger size and flexibility. Overall, the binding energy analysis shows that imprinted PPy has a higher affinity for the template, while the analyte size, conformer change, and flexibility drastically influence its recognition affinity, leading to wide or narrow distributions.

The comparison of binding energies with previously discussed results reveals the correlation between electron transfer, binding energies, and the density of complexes. Higher binding affinities are observed alongside higher charge transfers in the more compact complexes. The MIP has shown diverse but improved binding affinities via different imprinted binding sites. Therefore, careful binding and rebinding are vital to ensure that the process is completed with enough time to bind.

#### Imprinting Efficiency

Nonimprinted polymer (NIP) serves as a corresponding reference polymer to evaluate the imprinting efficiency. NIP is produced under conditions identical to those of MIP but without the presence of a template during polymer formation. Therefore, the same oligomers were used and further optimized with the same level of theory in the absence of a clorsulon template to form a PPy complex with its counterions. Then, analytes were placed onto the PPy chains and optimized by applying constraints to the heavy atoms of PPy. The comparison of binding energies exposed elevated binding energies for all clorsulon, AMX, and SA. The ratio of binding energies of MIP (average of binding sites) over NIP was employed as the measure of the imprinting factor (IF) (Figure 8C). Furthermore, fingerprint plots are presented to illustrate the enhanced surface area and NCI interactions in the MIP compared to the NIP, as shown in Figure 8A,B, respectively. The fingerprints are obtained by eq 9 with sampled spikes in a plane. The fingerprint reveals the close hydrogen donor-accepting atoms for H-bond formation between two fragments, the template and the binding sites. H-bond acceptors exhibit spikes with *x*-axis values greater than *y*-axis values where d_i_ > d_e_. H-bond-donating spikes appear in the axis regions with d_i_ < d_e_. The purple, green, and yellow colors correspond to low, medium, and high point densities, respectively. The overall visual comparison reveals a drastic increase in the surface area between the fragments. New spikes are observed at closer distances (*d*) where stronger NCI occurs. Figure 8D shows the Hirshfeld surface of clorsulon exposed to NIP, which corresponds to its fingerprint (Figure 8 B). The surface area is 200.62 Å^2^, the volume is 1197.17 Å^3^, and the density is 2.27 g/cm^3^. The values demonstrate that the modeled NIP has a much lower density compared to all binding sites provided in Table 2. The optimized PPy oligomers show a layered structure due to the extensive π–π stacking. The stacked PPy increases the rigidity and crystallinity of the polymer, while the presence of the template can reduce it.

**Figure 8:**
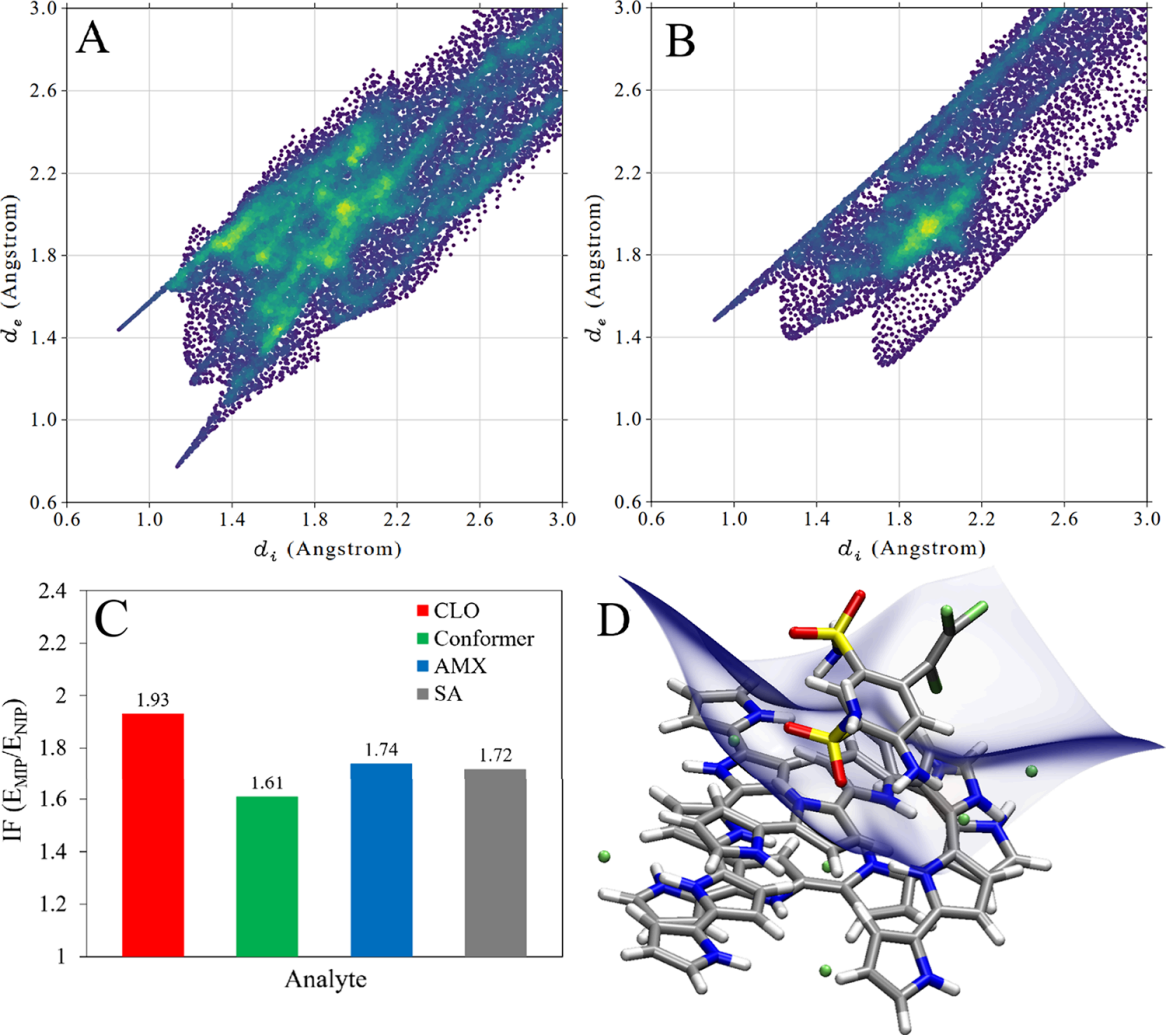
(A) Fingerprint plot of the first conformer of clorsulon (CLO1) onto BS1. (B) Corresponding nonimprinted PPy. (C) Estimated IF for the analytes. (D) Hirshfeld surface between clorsulon and nonimprinted PPy.

## Conclusions

Optimizing the prepolymerization step is essential for obtaining an imprinted polymer tailored to the target analyte. This study presented the in silico design and theoretical optimization of PPy imprinted with clorsulon. The findings demonstrate the optimal molar ratio of Py to clorsulon to be 16:1. The theoretical evaluation of the solvent effect on interactions in the prepolymerization mixture was demonstrated. The obtained results allow us to conclude that water should be selected as the least-interfering medium in the interactions within the prepolymerization mixture.

In this study, we demonstrate that a theoretical evaluation of conformers and rotamers is exceptionally important for predicting the efficiency of hosting properties, thereby enhancing binding affinities toward template molecules. Therefore, the imprinted region was modeled and presented with different conformers and rotamers that exhibit efficient hosting properties. The obtained results demonstrate that the PPy coupling type alters the physicochemical properties, as the folded chain formed by ββ and αβ linking reduces conjugation and increases flexibility. The clorsulon molecule exhibited conformer changes within 1 kcal mol^–1^, and later rebinding exposed the adverse impact of conformer changes on the binding energies of imprinted PPy at the binding sites. The sensing of clorsulon by PPy-based MIP was found to occur through electron back-donation of clorsulon and partial reduction of bipolaron PPy.

### Theoretical Methods and Computational Details

In the modeling of the prepolymerization step, three complex sets were considered to calculate binding energies using the Hartree–Fock method with the three empirical corrections (HF-3c). HF-3c is a fast HF method used for NCI energy studies in large systems. It includes corrections for short-ranged basis set incompleteness, dispersion corrections (DFT-D3) with the Becke–Johnson damping, and geometrical counterpoise (gCP) for basis set superposition error (BSSE) effects.^44^ In the next steps, the global hybrid meta-generalized-gradient approximation (mGGA) functional, r2SCAN0 (25% Hartree–Fock exchange),^45^ was used in the DFT calculations. The selection of the hybrid functional was made to avoid overdelocalization of electrons, which are present in pure mGGA functionals and reduce self-interaction error (SIE). This enables more accurate calculation of the electrochemical events, such as doping of PPy oligomers, band gaps, anion energies, and interactions. The use of the mGGA functional provides a better description of electronic properties than GGA by inclusion of kinetic energy density, so that geometries and energies are more accurate. The triple ζ basis set def2-mTZVPP with the auxiliary basis set def2-mTZVPP/J^46^ for molecules and def2-TZVPPD basis for counterions^47^ were implemented in the calculations. The basis sets were designated to account for the polarization of the electron density in molecules with two sets of polarization functions and an additional set of diffuse functions for Cl^–^ counterions. A tight SCF iteration was considered, with energy tolerances between two cycles of 1 × 10^–8^, RMS density changes of 5 × 10^–9^, and maximum density changes of 1 × 10^–7^. The atom-pairwise D4 scheme was applied to account for the London dispersion correction based on partial charges.^44^ The semilocal functionals do not account for long-range electron correlation, which is necessary to be treated for the molecular systems. All calculations were performed using free ORCA 6 software.^48,49^ The initial oligomer structure was derived using Avogadro 1.2.^50^ The initial structure coordinates of clorsulon and amoxicillin conformers were obtained from the PubChem database.^51^

The binding energies were calculated using eq 1:

$$E_{\mathrm{Binding}}=E_{\mathrm{Complex}}-\left(E_{\mathrm{Template}}+E_{\mathrm{Receptor}}\right)$$ 1

The solvation effect on the prepolymerization mixture was evaluated using the solvation model based on the molecular electron density (SMD) implicit model^52^ and the explicit solvent placement with the ORCA 6 Solvator Module, which uses the Automated Docking Algorithm.^16^ The SMD model is a robust implicit model that characterizes the solvent effect with more descriptors including electrostatics and cavity creation. The free energy of solvents is calculated by eq 2 for the SMD model as the sum of contributions of electrostatics, denoted as electronic nuclear polarization (*G*_ENP_), and short-range interactions by the cavity-dispersion solvent-structure *G*_CDS_ term. eq 3 was used for the explicit model with the semiempirical GFN2-xTB­(ALPB) parameters.^53^ The free energy of solvation (*G*_sol_) is the sum of electrostatic (*G*_elect_), solvent access surface area (*G*_SASA_), and H-bonds (*G*_H‑bond_). Δ*G*_shift_ is a constant shift that depends on the reference state for the solvation process.

$$G_{\mathrm{sol}}=\Delta G_{\mathrm{ENP}}+\Delta G_{\mathrm{CDS}}$$ 2

$$G_{\mathrm{sol}}=G_{\mathrm{elec}}+G_{\mathrm{SASA}}+G_{\mathrm{Hbond}}+G_{\mathrm{shift}}$$ 3

Crest 3.0.2 software was used to perform iMTD-GC for conformational search.^54^ This method enables conformer and rotamer searches using the RMSD-based collective variable applied in the parallel metadynamics simulations, combined with the GFN2-xTB level of calculation. The metadynamics durations were automatically set by the software based on the flexibility measure. The multilevel ensemble optimization method with tight thresholds and the shake algorithm were applied to all bonds to optimize the obtained structures in the produced trajectories.

Jmol 16.2.17 software was used to generate the MEP of molecules within a ± 0.07 potential range.^55^ The BLA^56,57^ for conjugation analysis was calculated using eq 4:

$$\mathrm{BLA}=\frac{1}{N}\sum_{i=1}^{N}|L_{i}-L_{i+1}|$$ 4

where *L* is the length of the bond (*i*), and *N* is the number of the double–single bond pairs.

HOMO and LUMO energies (ε) were obtained in (eV), and the band gap (*E*_g_) was calculated with *E*_g_ = ε_LUMO_ – ε_HOMO_. Then, using Koopman’s theorem, IP from IP = −ε_HOMO_ and EA from EA = −ε_LUMO_ were derived.^58^

The chemical potential (μ) and the hardness (η) were calculated from eqs 5 and 6, respectively.

$$\mu =\frac{\mathrm{IP}+\mathrm{EA}}{2}$$ 5

$$\eta =\frac{\mathrm{IP}-\mathrm{EA}}{2}$$ 6

The global electrophilicity (ω) index and the maximum charge transfer (Δ*N*_max_) were obtained using eqs 7 and 8.^59^

$$\omega =\frac{\mu^{2}}{2\eta }$$ 7

$$\Delta N_{\max}=\frac{-\mu }{\eta }$$ 8

Multiwfn 3.8 software^60,61^ was used to analyze the data obtained from optimized molecular systems. Hirshfeld surface analysis was performed based on electron density to determine the size, shape, volume, and density of binding sites (eq 9).^62^

$$d_{\mathrm{norm}}=\frac{d_{i}-r_{i}^{\mathrm{VDW}}}{r_{i}^{\mathrm{VDW}}}+\frac{d_{e}-r_{e}^{\mathrm{VDW}}}{r_{e}^{\mathrm{VDW}}}$$ 9

where *d*_norm_ is the normalized contact distance, and *d*_i_ and *d*_e_ are the distances from a point on the surface to the nearest nucleus inside and outside the surface, respectively. *r* ^VDW^ is the van der Waals (VDW) radius of the corresponding two atoms.

The RDG is derived from the electron density and its first derivative using eq 10. Multiwfn was used for NCI analysis for the qualitative study of inter- and intramolecular NCI. In this analysis, sign­(λ_2_)­ρ is the sign of the second largest eigenvalue of the electron density, Hessian matrix (λ_2_), in electron density (ρ). This function, together with RDG, is used to study weak interactions.

$$\mathrm{RDG}(r)=\frac{1}{2(3\pi^{2}{)}^{1/3}}\frac{\left|\nabla \rho (r)\right|}{{\rho (r)}^{4/3}}$$ 10

VMD 2.0 software was used to visualize the Hirshfeld and RDG surface.^63^ The interactions in the complex were produced by BIOVIA Discovery Studio Visualizer.^64^ The DOS was obtained using the Hirshfeld method. PDOS of PPy and Cl^–^ counterions, as well as clorsulon, were obtained using Multiwfn 3.8 software. CDA^65^ was carried out using the modified Mulliken population analysis (SCPA) method. This analysis aims to provide insights into how charges are transferred between fragments, the binding site, and the template molecule in the complex, to reach charge equilibrium and to visualize the MO compositions.^66^ The CDA is based on the fragment orbital (FO), which signifies MOs of each fragment building the complex in its isolated state. Time-dependent DFT was performed with the Tamm–Dancoff approximation (TD-DFT/TDA) with inclusion of singlet and triplet excitations in the C-PCM water solvent.

## Supplementary Material



## Notes

Summary of the employed computational methods, clorsulon conformer change during AIMD simulation, RDG isosurface of binding sites complexed with rebound clorsulon template, MO interaction diagrams for the BS1-CLO1 complex, and optimized structures of AMX conformers and SA (PDF)

## List of Abbreviations

AIMD: ab initio molecular dynamics
AMX: amoxicillin
BSSE: basis set superposition error
BS: binding site
BLA: bond length alternation
CDS: cavity-dispersion solvent structure
CDA: charge decomposition analysis
CLO: clorsulon
DFT: density functional theory
EA: electron affinity
FO: fragment orbital
gCP: geometrical counterpoise
HF-3c: Hartree–Fock method with three empirical corrections
IF: imprinting factor
IP: ionization potential
KDE: kernel density estimation
LOL: localized orbital locator
MTD: metadynamics
mGGA: meta-generalized gradient approximation
MEP: molecular electrostatic potential
MO: molecular orbital
MIP: molecularly imprinted polymer
NCI: noncovalent interactions
NIP: nonimprinted polymer
PDOS: partial density of states
PCM: polarizable continuum model
PPy: polypyrrole
Py: pyrrole
RDG: reduced density gradient
RMSD: root-mean-square deviation
SA: salicylic acid
SASA: solvent-accessible surface area
TDOS: total density of states
VDW: van der Waals
xTB: extended tight binding.
