# Supplementary material for: A Theoretical Study of Clorsulon-Imprinted Polypyrrole: Modeling Complementary Cavity Formation and Rebinding of Clorsulon
Source: ACS Meas Sci Au. 2026 May 8;6(3):811–24. doi: 10.1021/acsmeasuresciau.6c00052 (PMC13281173; doi:10.1021/acsmeasuresciau.6c00052)
Supplement: Supplementary file 1 [file tg6c00052_si_001.pdf]

Supplementary material for:

A Theoretical Study of Clorsulon Imprinted Polypyrrole: Modelling Complementary Cavity Formation and Rebinding of Clorsulon

Enayat Mohsenzadeh [1], Vilma Ratautaite [1, *], Agne Ramanaviciute[2,3], Arunas Ramanavicius [1, 2 *]

[1] Department of Nanotechnology, State Research Institute Centre for Physical Sciences and Technology (FTMC), Sauletekio Ave. 3, LT-10257 Vilnius, Lithuania; enayat.mohsenzadeh@ftmc.lt; vilma.ratautaite@ftmc.lt; arunas.ramanavicius@chf.vu.lt.

[2] Department of Physical Chemistry, Institute of Chemistry, Faculty of Chemistry and Geosciences, Vilnius University (VU), Naugarduko Str. 24, LT-03225 Vilnius, Lithuania;

[3] Department of Physics, University of Cambridge, JJ Thomson Avenue, CB3 0HE, Cambridge, United Kingdom.

Corresponding authors: Dr Vilma Ratautaite (vilma.ratautaite@ftmc.lt); Prof. habil. Dr Arunas Ramanavicius (arunas.ramanavicius@chf.vu.lt)

PhD Student Enayat Mohsenzadeh	https://orcid.org/0000-0002-6598-7622
Dr. Vilma Ratautaite	https://orcid.org/0000-0002-9931-4397
PhD Student Agne Ramanaviciute	https://orcid.org/0009-0007-6999-4516
Prof. habil. dr. Arunas Ramanavicius	https://orcid.org/0000-0002-0885-3556

A 2000 fs, 1 fs time-step AIMD simulation of Clorsulon showed that the functional rotation of sulphonamide is far from being the most feasible conformational change in water solvent. The same level of theory (r2SCAN0/def2-mTZVPP) with the D4 correction was used. The Berendsen thermostat was implemented with a 300 K temperature target.

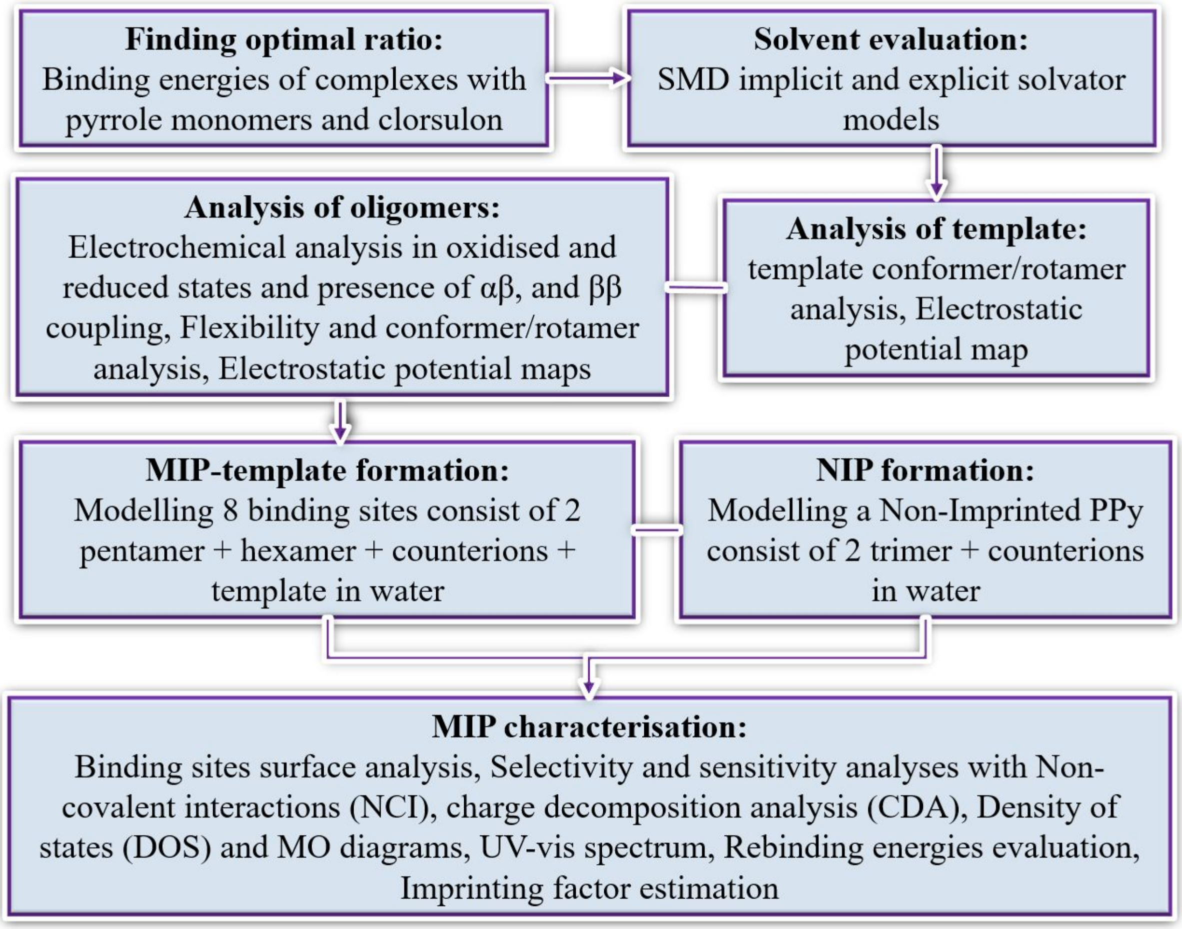

Figure S1. Summary of implemented computational methods for PPy-based MIP evaluation as a clorsulon sensor.

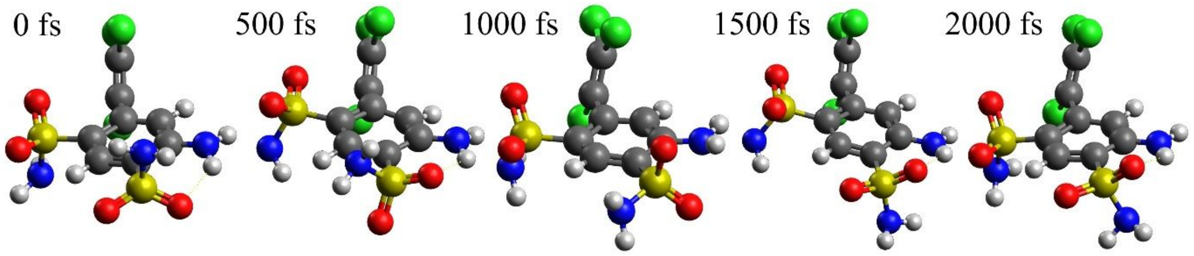

Figure S2. Conformer change of clorsulon over time in 2 ns.

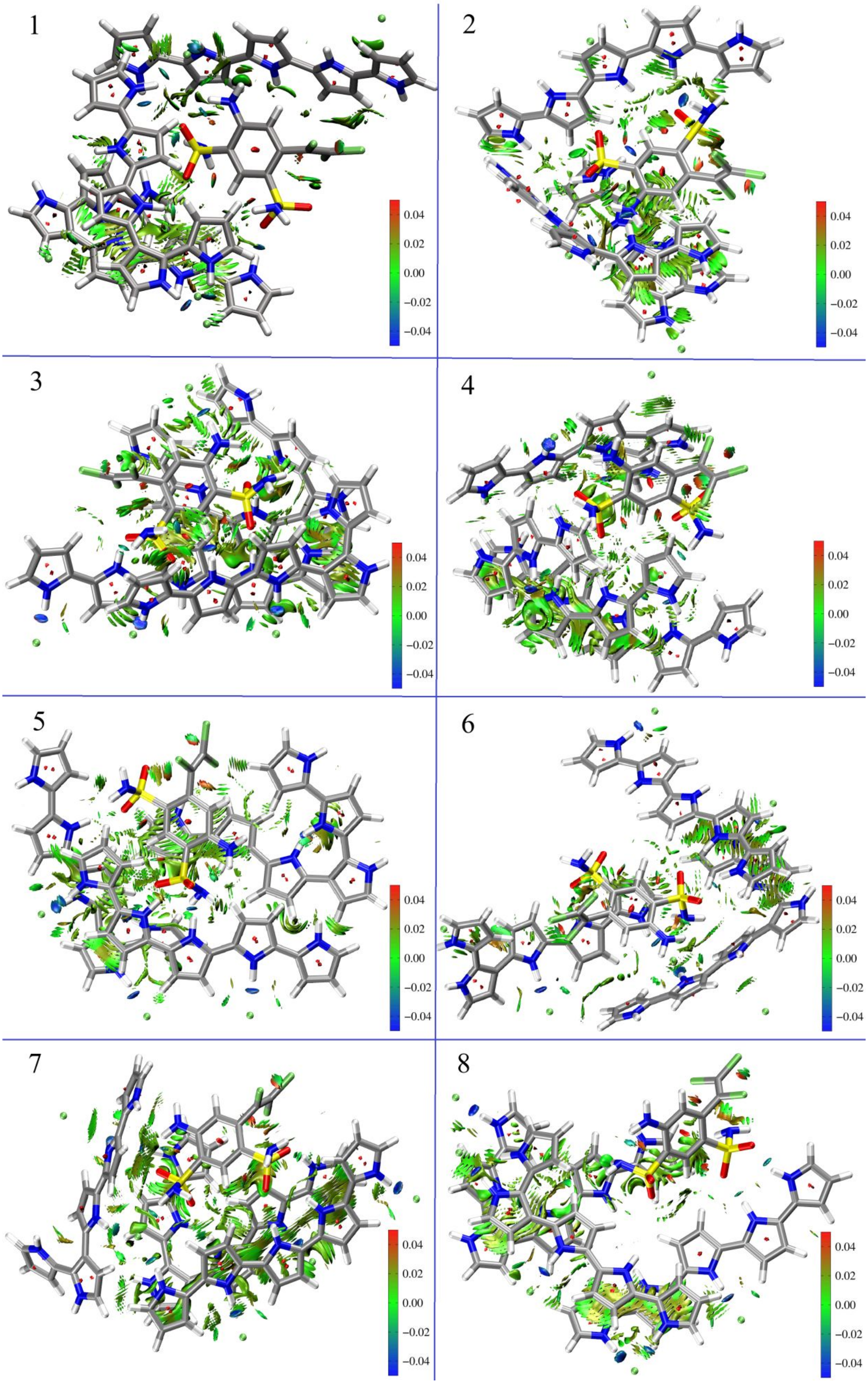

Figure S3. RDG isosurface of binding sites 1-8 displays the non-covalent interactions.

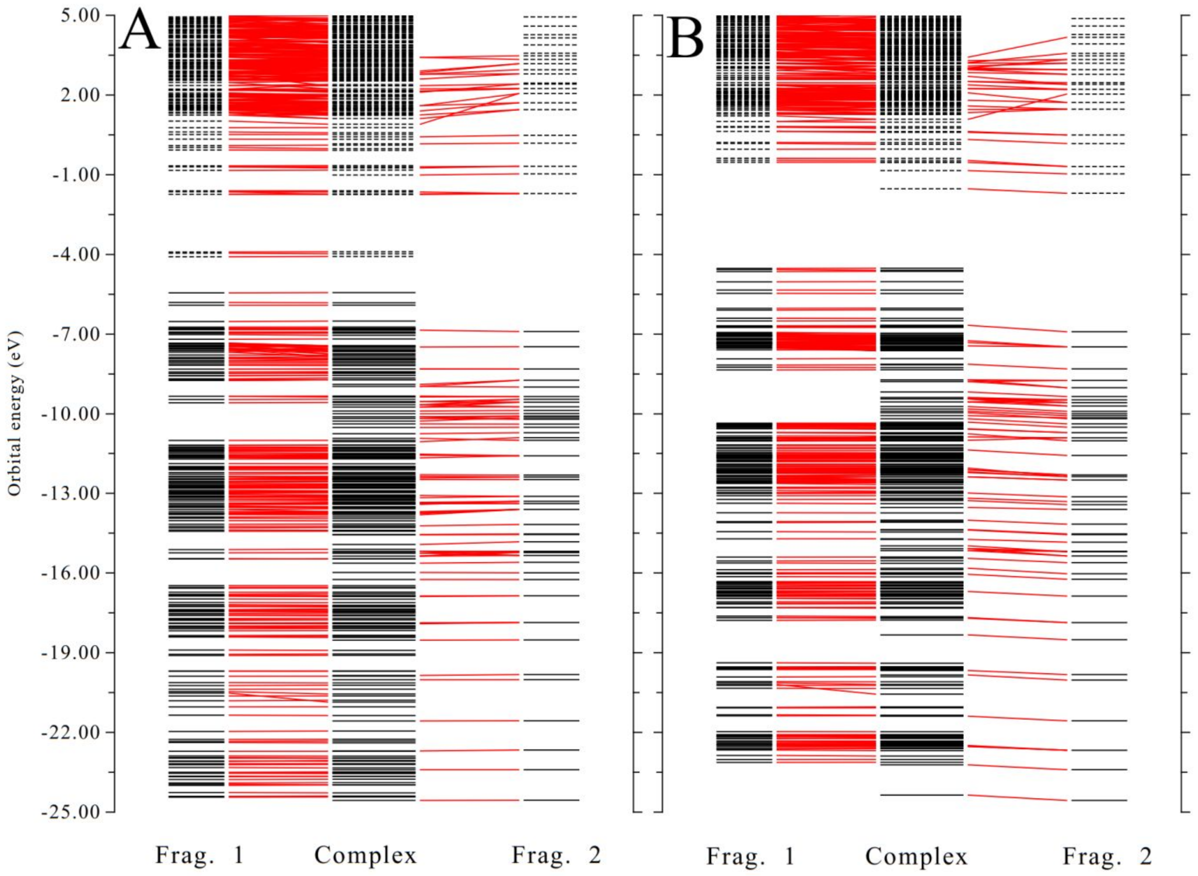

Figure S4. (A) MOs interaction diagrams for binding site 1 (BS1) in the oxidised state complexed with CLO; **(B)** MOs interaction diagrams for binding site 1 (BS1) in the reduced state complexed with CLO. Occupied and virtual orbitals are represented by solid and dashed bars, respectively. The bars in the middle correspond to complex orbitals

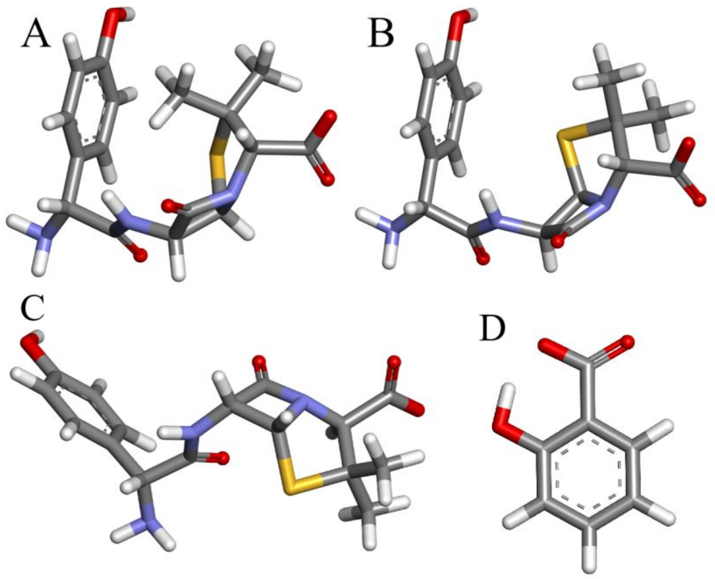

Figure S5. (A, B, and C) Amoxicillin (AMX) conformers, and **(D)** salicylic acid (SA) in the deprotonated state were optimised in the implicit SMD water solvent.
